# Assessment of food specific immunoglobulin load in pre-school children

**DOI:** 10.1186/1939-4551-8-S1-A54

**Published:** 2015-04-08

**Authors:** Vithal Jathar

**Affiliations:** 1 Alletess Medical Laboratory, USA

## Background

Chronic diarrhea, vomiting, reflux, bloating, flatulence, and chronic stomach pain accompanied by cranky behavior are common symptoms related to delayed food sensitivity reactions in preschool children ranging from one to five years of age. It is not known whether immune intolerance and/or inflammatory reactions are playing any role in these children due to over-reactivity to food specific immunoglobulin load.

## Methods

At Alletess Medical Laboratory, we analyzed 966 preschool children. Our 96 food ELISA assay was used to assess food specific IgG antibody loads at three different levels low (10 – 25 ug/ml), moderate (25 – 50 ug/ml), and high (>50 ug/ml). The 96 foods were categorized into dairy products, animal proteins, grains, nuts, fish, shell fish, herbs, spices, vegetables, and fruits groups.

## Results

Our results revealed the following percentages of this subpopulation with high IgG antibody loads: dairy products (18 - 21%), grain/seeds (19 - 32%), eggs (29%), tree nuts (6 - 20%), peanuts (29%), animal proteins (< 2%), vegetables and fruits (< 5%), and yeasts (3 - 13%).

## Conclusions

Overload of food specific IgG antibodies could be the cause of intolerance and/or symptoms related to inflammatory reactions of the bowels. The burden related to this IgG overload could be lowered by replacing offending foods with well-balanced food alternatives in preschool children in order to improve their overall wellness.

Further clinical studies are recommended to confirm these findings including observing IgA and IgE immunoglobulin levels as well as IL-10 level studies for those with bowel related symptoms.

